# Molecular Dynamics Study of Wetting and Adsorption of Binary Mixtures of the Lennard-Jones Truncated and Shifted Fluid on a Planar Wall

**DOI:** 10.1021/acs.langmuir.1c00780

**Published:** 2021-06-07

**Authors:** Michaela Heier, Simon Stephan, Felix Diewald, Ralf Müller, Kai Langenbach, Hans Hasse

**Affiliations:** † Laboratory of Engineering Thermodynamics, Technische Universität Kaiserslautern, 67663 Kaiserslautern, Germany; ‡ Institute of Applied Mechanics, Technische Universität Kaiserslautern, 67663 Kaiserslautern, Germany; § Thermal Separation Science (Endowed Professorship of the State Tyrol), University of Innsbruck, 6020 Innsbruck, Austria

## Abstract

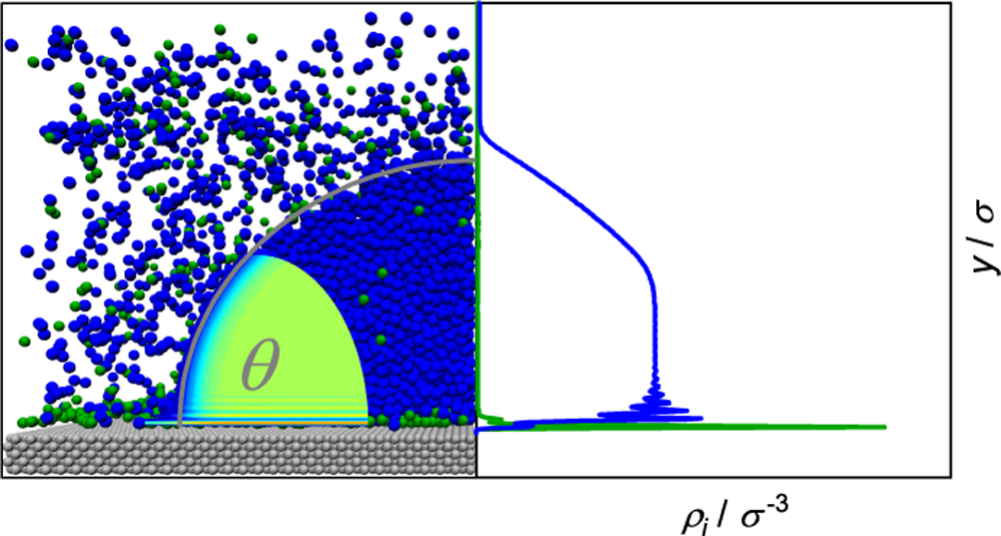

The wetting of surfaces is strongly influenced by adsorbate layers. Therefore, in this work, sessile drops and their interaction with adsorbate layers on surfaces were investigated by molecular dynamics simulations. Binary fluid model mixtures were considered. The two components of the fluid mixture have the same pure component parameters, but one component has a stronger and the other a weaker affinity to the surface. Furthermore, the unlike interactions between both components were varied. All interactions were described by the Lennard-Jones truncated and shifted potential with a cutoff radius of 2.5σ. The simulations were carried out at constant temperature for mixtures of different compositions. The parameters were varied systematically and chosen such that cases with partial wetting as well as cases with total wetting were obtained and the relation between the varied molecular parameters and the phenomenological behavior was elucidated. Data on the contact angle as well as on the mole fraction and thickness of the adsorbate layer were obtained, accompanied by information on liquid and gaseous bulk phases and the corresponding phase equilibrium. Also, the influence of the adsorbate layer on the wetting was studied: for a sufficiently thick adsorbate layer, the wall’s influence on the wetting vanishes, which is then only determined by the adsorbate layer.

## Introduction

The wetting of solids plays an important role in many processes. It is usually characterized by the contact angle θ of a sessile drop on a surface and depends on the interactions between all components of the investigated system.

Technical surfaces are always contaminated by adsorbed residues, which form an adsorbate layer. The composition and thickness of that adsorbate layer depend on the pretreatment of the surface, the surrounding fluid (e.g., air), and the underlying substrate and can, for example, be studied by X-ray photoelectron spectroscopy (XPS).^1,2^ Surfaces without adsorbate layers (i.e., atomically clean surfaces) can only be obtained by special treatments such as plasma cleaning and storage in ultrahigh vacuum. The adsorbate layer leads to a change in the wetting behavior compared to the atomically clean surface^3^ and it has long been known that the adsorbate layer strongly influences the contact angle of a sessile drop and must not be neglected in studies of wetting of surfaces.^4^ In most situations, the underlying substrate has no direct influence on the sessile drop when the adsorbate layer is thicker than about 1–2 nm.^5,6^ In recent experimental studies on surfaces with adsorbate layer, that is, gold, steel, and titanium, Heier et al.^2^ observed that the contact angle depends only on the adsorbate layer composition for an adsorbate layer thicker than 1.4 nm.^1,2^ In contrast, the wetting is influenced by the underlying substrate directly when the adsorbate layer is thinner than about 1 nm.^7^

Molecular simulations help gaining a detailed understanding of wetting phenomena and have been carried out by many authors, see for example, refs (8−10) Molecular simulation studies of surface wetting by a pure fluid obviously describe the wetting of atomically clean surfaces. To describe the influence of the adsorbate layers on the wetting, fluid mixtures have to be studied. This is done here in a systematic manner using model mixtures.

Wetting transitions, that is, prewetting or demixing, on planar walls have been studied previously by many authors using molecular simulations.^11−20^ Investigations of sessile drops of binary mixtures on planar walls have been carried out during the last 10 years by several groups.^21−29^ Seveno et al.,^21,22^ Das and Binder,^23,24^ Jiang et al.,^25^ and Surblys et al.^26^ focused on methods for predicting the contact angle from the surface tensions, whereas Kumar and Errington^27,28^ describe methods for obtaining the contact angle by spreading and drying coefficients from Monte Carlo simulations. Seveno et al.,^21,22^ Das and Binder,^23,24^ Jiang et al.,^25^ and Kumar and Errington^27,28^ investigated the contact angle on a solid in a system with two immiscible liquids, whereas Surblys et al.^26^ investigated the wetting of water–methanol or water–isopropyl alcohol mixtures with different alcohol mass fractions on a solid wall with molecular dynamics (MD) simulations. Lundgren et al.^29^ investigated the wetting of a water–ethanol droplet on a solid graphite surface with MD simulations for different mole fractions of ethanol.

In contrast to these previous studies, we systematically investigated the influence of the adsorbate layer on the wetting. Thereby, binary fluid mixtures with varying unlike fluid–fluid interactions were used. Furthermore, the attraction of the wall differs for the two components of the mixtures, which leads to a different adsorption of the two components.

In recent MD simulation studies, we have investigated the wetting and the adsorption of pure fluids on planar walls with the Lennard-Jones truncated and shifted (LJTS) potential with a cutoff radius of 2.5σ.^30,31^ The same potential is used in the present work. The LJTS potential describes properties of simple fluids for a wide range of states and its properties are well known,^30−44^ both for pure fluids and mixtures, and it has been used as a model fluid for many studies, for example, see refs (30−39, 45−55). The LJTS potential gives only crude descriptions of solids. However, as the present study does not focus on the solid itself but rather on the influence of the solid–fluid interactions on the wetting and adsorption, we use the LJTS potential also for the solid, for simplicity.

In the present work, the wetting of a planar LJTS wall by different binary LJTS fluid mixtures is investigated. The two components of the fluid had the same pure component parameters, only the parameter describing the unlike dispersive interactions was varied such that different types of fluid mixture behavior were obtained: a mixture with a (low-boiling) hetero-azeotrope, an ideal mixture, and a mixture with a high-boiling azeotrope. Furthermore, one of the fluid components was attracted more strongly by the solid than the other. The temperature was kept constant for all simulations conducted in the present work.

From the simulation results, information on different properties was obtained: the adsorption (surface excess, structure and composition, and layer thickness), the contact angle, and bulk data of the liquid drop and the surrounding vapor phase. Cases with total wetting and cases with partial wetting were observed.

The paper is organized straightforwardly: first, the molecular model and simulation method are described, followed by the description of the evaluation of the simulation data. Then, the results are presented and discussed and the conclusions are drawn.

## Experimental Section

In our work, only computer experiments were conducted.

### Molecular Simulation

#### Molecular Model

In this work, the LJTS 12-6 potential *u*_LJTS_ was used for describing the interactions between all particles. It is based on the Lennard-Jones (LJ) 12-6 potential *u*_LJ_
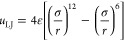
1
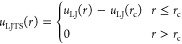
2with ε and σ as the energy and size parameter, respectively, and *r* as the distance between two particles.^56^ The LJTS potential was truncated and shifted at a cutoff radius *r*_c_ of 2.5σ throughout the present work.

The size parameter σ and the mass *m* of all fluids and the solid were the same. Binary fluid mixtures consisting of two identical fluid (f) components A and B were studied, that is, not only the size parameters but also the energy parameters ε_f_ of the fluids were the same.

**Figure 1:**
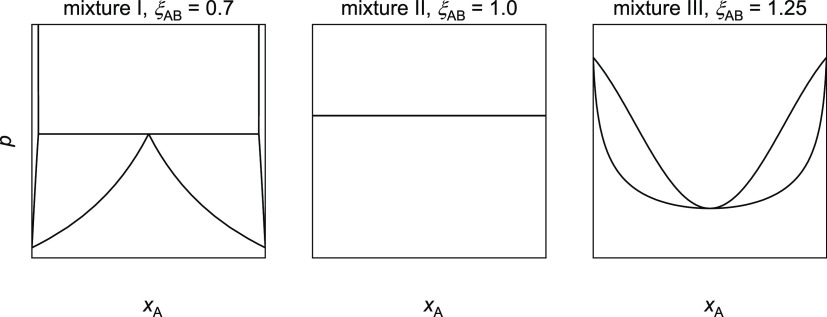
Sketches of the isothermal *p*–*x* phase diagrams of the binary LJTS fluid mixtures studied in the present work. The pressure *p* is plotted versus the mole fraction of component A *x*_A_ in the bulk phases. The pure fluid components A and B are the same, but the binary interaction parameter ξ_AB_ is varied. For ξ_AB_ = 1.0, the vapor-liquid equilibrium region degenerates to a line, for ξ_AB_ = 0.7, there is a (low-boiling) hetero-azeotrope, whereas for ξ_AB_ = 1.25, there is a high-boiling homogeneous azeotrope.

The unlike fluid–fluid interactions were described using the modified Lorentz–Berthelot combination rules^57,58^ for the binary interaction energy and size parameter
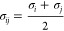
3
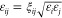
4where the indices *i* and *j* indicate the components and ξ_*ij*_ is the binary interaction parameter. Equation 3 is only provided for completeness; the size parameter σ was the same for all interactions. The binary interaction parameter ξ_*ij*_, however, was varied. Three fluid mixtures A + B were considered. They vary in the binary interaction parameter ξ_AB_, for which the numbers were: ξ_AB_ = 0.7, 1.0, and 1.25. The unlike fluid–fluid interactions are unfavorable for ξ_AB_ = 0.7, ideal for ξ_AB_ = 1.0, and favorable for ξ_AB_ = 1.25. These binary interaction parameters lead to a mixture with a vapor–liquid–liquid equilibrium (VLLE) and a miscibility gap (mixture I), an ideal mixture (mixture II), and a mixture with a high-boiling azeotrope (mixture III). Sketches of the phase behavior of the three mixtures are shown in Figure 1.

The energy parameter of the solid (s) was ε_s_ = 100ε_f_ and the solid wall had a crystal configuration with a face-centered cubic lattice with the (100) surface exposed to the fluid. The crystal configuration remained unchanged during the simulation due to the high energy parameter of the solid. The present choices lead to a lattice constant *a* = 1.55σ and a density of the solid of ρ_s_ = 1.07σ^–3^.

The binary fluid mixtures interacted with the solid wall, whereas fluid component A was attracted more strongly (ξ_sA_ = 0.10) than fluid component B (ξ_sB_ = 0.035). The wetting behavior of the pure fluids A and B on the wall is known from a previous study of Becker et al.^30^ and can be calculated by a correlation. This correlation leads to total wetting for component A (θ_A_ = 0°) and to partial wetting for component B (θ_B_ = 123.8°) at the studied temperature.

Throughout the present work, all properties are normalized using the Boltzmann constant *k*_B_, the mass *m*, the size parameter σ, and the energy parameter ε_f_ of the fluid.

#### Simulation Method

**Figure 2:**
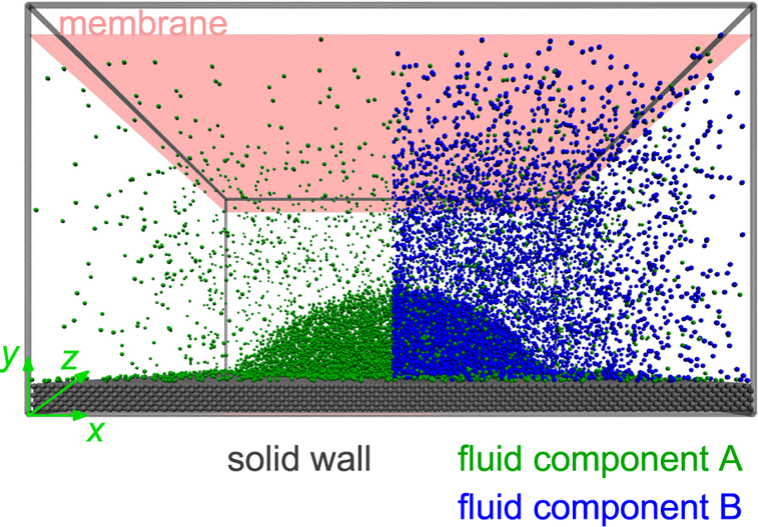
Snapshot of the wetting scenario with a binary fluid mixture. On the right side, the fluid components A and B are depicted, whereas on the left side, only the fluid component A is depicted.

For investigating the wetting and adsorption of the binary fluid mixtures, MD simulations were carried out in the canonical (*NVT*) ensemble with the massively parallel program *ls1 mardyn*.^52^ A snapshot of the simulation scenario used in this work is shown in Figure 2. As in our previous study,^31^ the scenario contained an atomistic wall, which was composed of six layers of LJTS sites. The atomistic wall was located in the *x*,*z*-plane of a Cartesian coordinate system and the *y*-coordinate was perpendicular to the wall’s surface. It was fixed at the bottom of the simulation box by applying an external potential on the lowest layers of the solid, as described in detail in the Supporting Information. Periodic boundary conditions were applied in all directions. To avoid fluid layer growth underneath the wall, a repulsive soft membrane with a reset force of *F* = −20ε_f_σ^–2^·Δ*y* was applied at *y* = 65σ with Δ*y* as the distance from particles above the membrane to the membrane. Even for large contact angles of the droplet, the distance between the droplet and the membrane was sufficiently large to avoid any influence of the membrane on the droplet. The size of the simulation box was the same for all simulations: the height of the simulation box was *L*_*y*_ = 70σ, whereas the width and the depth were *L*_*x*_ = *L*_*z*_ = 125σ.

All simulations started with a hemispherical liquid droplet (contact angle θ = 90°) on top of the wall in the middle of the *x*,*z*-plane surrounded by a vapor phase. The fluid particles of the liquid and the vapor phase were initialized on a lattice, whereat the liquid phase had a high density and the vapor phase a low density. The initial composition of the fluid mixtures was the same for the vapor and the liquid phase. During equilibration, the composition of the fluid mixtures in the vapor and liquid phase changes, as well as the liquid contact angle. Particles are attracted by the wall and form an adsorbate layer. This equilibration process may lead to high particle velocities and, as a consequence, to an instability of the droplet. Preliminary studies showed that these problems can be circumvented by lowering the temperature in the first equilibration steps. Therefore, a constant temperature of *T* = 0.65ε_f_*k*_B_^–1^ was chosen for the first 3.5 million time steps. After 3.5 million time steps, the temperature was set to *T* = 0.75453ε_f_*k*_B_^–1^, corresponding to 0.7*T*_c_ (with the critical temperature *T*_c_ = 1.0779ε_f_*k*_B_^–1^).^32^ The equilibration time is determined by the equilibration of the adsorbate layer and the liquid droplet and it is much longer than for pure fluids, cf. refs (30,31). At least 13 million time steps were used. The equilibration was followed by a production time of 2.5 million time steps. The time step was Δ*t* = 0.0005(*m*/ε_f_)^1/2^σ.

During the simulation, the temperature was kept constant individually for each of the fluids and the solid by an Andersen thermostat^59^ with a collision frequency of ν = 0.05. The total number of fluid particles varied between 61,000 and 90,000 such that a sufficient number of particles of both components was present in the simulations to obtain acceptable statistics for all fluid regions, cf. refs (30,31). The number of wall particles was constant for all simulations (*N*_s_ = 77,760).

#### Data Evaluation

**Figure 3:**
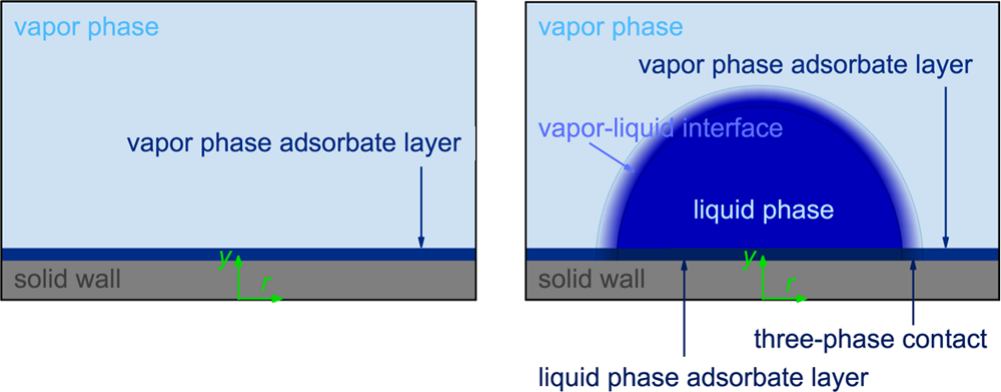
Spatial regions that were distinguished in the evaluation of the simulation results for the cases of total wetting (left) and partial wetting (right).

Depending on the settings, two different cases were observed in the present simulations: total wetting and partial wetting. In the evaluation of the simulations, different regions were distinguished, which are illustrated in Figure 3 for these two cases. In both cases, there is a vapor phase and an adsorbate layer at the wall below that vapor phase, which is called vapor phase adsorbate layer in the following. For partial wetting, additionally, the following regions were distinguished: the bulk liquid droplet with the liquid phase adsorbate layer below, the vapor–liquid interfacial region, and the three-phase contact. The differentiation of these regions is explained in more detail below. In the present work, the following quantities were measured in the stated regions:

- vapor phase (bulk): the component densities ρ_A_^″^ and ρ_B_^″^, the total pressure *p*″, and the mole fraction of component A *x*_A_^″^
- vapor phase adsorbate layer: the average mole fraction of component A *x*_A_^″,ads^, the surface excess of both components Γ_A_^″^ and Γ_B_^″^, and the adsorbate layer thickness of both components δ_A_^″^ and δ_B_^″^
- liquid phase (bulk): the component densities ρ_A_^′^ and ρ_B_^′^ and the mole fraction of component A *x*_A_^′^

For the case of partial wetting, the contact angle θ and the droplet radius *R*_d_ were also measured. The liquid phase adsorbate layer was not evaluated quantitatively in the present work because the quantities of the liquid phase adsorbate layer were influenced by the vapor–liquid interface for small droplets and could not be measured with sufficient accuracy. The vapor–liquid interface as well as the three-phase contact were also not studied quantitatively in the present work. The vapor–liquid interface of binary LJTS mixtures was comprehensively studied in previous work of our group.^36,46,60−62^ The total pressure in the liquid phase differs from that calculated for the vapor phase by the pressure difference for small droplets resulting from the Young–Laplace equation^63^ and was not determined in the present work. Detailed information on the calculation of the quantities stated above are given in the Appendix.

For the evaluation of the simulation results, not the Cartesian coordinate system shown in Figure 2 was used but rather a cylindrical coordinate system. The *y*-axis of that coordinate system is parallel to the *y*-axis of the Cartesian system but goes through the symmetry axis of the droplet; cf. Figure 3. As in the Cartesian system, *y* = 0σ is at the lower end of the wall. The cylindrical coordinate system is convenient for the simulations with partial wetting and is basically the same as that used by Becker et al.^30^ in their work with simulations of droplets at walls. For consistency, the cylindrical coordinate system was also used for the evaluation of the simulations with total wetting. Due to the cylindrical coordinate system, the corners of the simulation box are not considered in the data evaluation.

Component density fields ρ_A_(*y*,*r*) and ρ_B_(*y*,*r*) were sampled as block average with a block size of 500,000 time steps during the simulation run via binning in the cylindrical coordinate system. 466 bins of equal size were used both in *y*- and *r*-direction. The density was sampled by counting the particles per bin. The density fields were used to determine the liquid phase quantities as well as the contact angle and the droplet radius by calculating the vapor–liquid interface of the droplet; cf. the Appendix.

For characterizing the vapor phase and liquid phase adsorbate layers, fluid component density profiles ρ_*i*_(*y*) with *i* = A, B were used here. These profiles were calculated by averaging the density fields ρ_*i*_(*y*,*r*) over *r*. For total wetting, the density fields were averaged over all *r*. For partial wetting, however, the influence of the three-phase region is excluded by 5σ in each direction. This results in vapor side density profiles ρ_*i*_^v^(*y*) for (*R*_d_ + 5σ) < *r* < 62.5σ and droplet side density profiles ρ_*i*_^d^(*y*) for 0 < *r* < (*R*_d_ – 5σ).

The droplet side density profiles were used in the present work to gain a qualitative insight into the liquid phase adsorbate layer; they were not used quantitatively. The calculation of the vapor phase bulk and vapor phase adsorbate layer quantities was based on the vapor side density profiles. They were also used to give qualitative insights into the vapor phase adsorbate layer.

**Figure 4:**
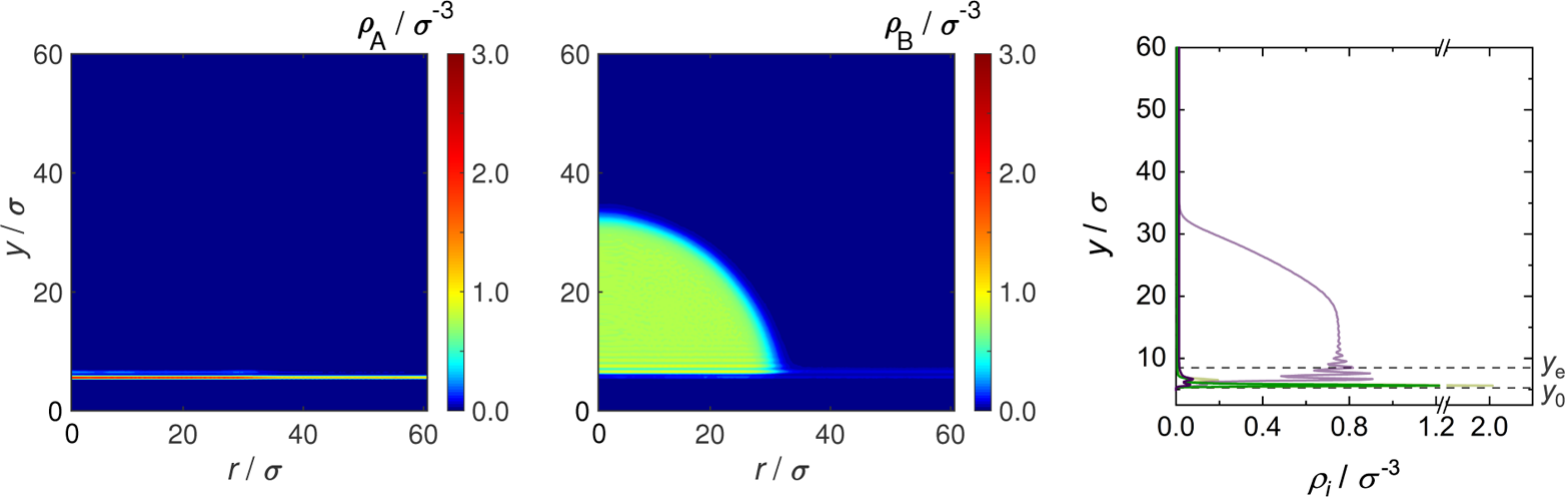
Fluid component density fields and profiles for ξ_AB_ = 0.7 and *x*_A_^″^ = 0.251 mol mol^–1^. Left: ρ_A_(*y*,*r*); middle: ρ_B_(*y*,*r*); right: ρ_A_^d^(*y*) (bright green), ρ_B_^d^(*y*) (bright violet), ρ_A_^v^(*y*) (dark green), and ρ_B_^v^(*y*) (dark violet). The lower integration boundary for the surface excess calculation *y*_0_ and the end value of the layer thickness calculation *y*_e_ are depicted as dashed gray lines.

Figure 4 shows exemplary density fields ρ_A_(*y*,*r*) and ρ_B_(*y*,*r*) and the corresponding density profiles ρ_A_(*y*) and ρ_B_(*y*) for a partial wetting case. All ρ_*i*_(*y*) show a layering at the wall. ρ_B_^d^(*y*) shows the layering of a liquid adsorption, followed by a plateau of the component density in the liquid droplet, which corresponds to the bulk liquid properties. For larger *y*, a smooth decrease to the component density in the vapor phase is observed. This decrease simply results from the averaging over *r* and the increasing amount of vapor phase in the considered volume as the *y*-coordinate approaches the droplet top. On the vapor side for ρ_A_^v^(*y*) and ρ_B_^v^(*y*), typical vapor phase adsorption density profiles are observed.

In this work, the uncertainty of the calculated quantities, that is, *x*_A_^″^, *x*_A_^′^, *x*_A_^″,ads^, *p*″, ρ_A_^″^, ρ_A_^′^, ρ_B_^″^, ρ_B_^′^, θ, Γ_A_^″^, Γ_B_^″^, δ_A_^″^, and δ_B_^″^, was estimated to be three times the standard deviation of five block averages of the production run (2,500,000 time steps). Fluid component density fields and profiles that are shown in the following were averaged over 2,500,000 time steps. The averaged fluid component density profiles and the corresponding uncertainties are given for all simulations in an Excel spreadsheet in the Supporting Information.

## Results and Discussion

In the present work, simulations for three fluid mixtures with different unlike fluid–fluid interactions and with varying composition of the fluid were carried out. The numerical results are given in Tables 1 and 2.

**Table 1:** Simulation Results in the Bulk Phases for Varying Unlike Fluid–Fluid Interaction Parameter ξ_AB_ and Number of Fluid Particles of the Components A (*N*_A_) and B (*N*_B_): Component Densities ρ_A_^″^ and ρ_B_^″^ in the Vapor Phase, Component Densities ρ_A_^′^ and ρ_B_^′^ in the Liquid Droplet, Vapor Phase Mole Fraction of Component A *x*_A_^″^, Liquid Phase Mole Fraction of Component A *x*_A_^′^, and Total Pressure *p*″ in the Vapor Phase^a^

ξ_AB_	*N*_A_	*N*_B_	ρ_A_^″^/σ^–3^	ρ_B_^″^/σ^–3^	ρ_A_^′^/σ^–3^	ρ_B_^′^/σ^–3^	*x*_A_^″^/mol mol^–1^	*x*_A_^′^/mol mol^–1^	*p*″/ε_f_σ^–3^
0.70	8920	80,285	0.00371(14)	0.01381(28)	0.0063(10)	0.7538(17)	0.2120(47)	0.0083(13)	0.0120(6)
	12,303	49,215	0.00477(28)	0.0142(5)	0.0079(13)	0.7521(28)	0.251(8)	0.0104(17)	0.0127(7)
	24,607	36,911	0.00889(42)	0.01441(37)	0.0152(25)	0.745(6)	0.381(10)	0.0200(33)	0.0154(5)
	36,910	24,608	0.0118(6)	0.01474(35)	0.023(6)	0.735(16)	0.444(11)	0.030(9)	0.0172(6)
	49,214	12,304	0.0128(8)	0.01093(41)			0.539(9)		0.0155(6)
1.00	8920	80,285	0.00104(10)	0.01212(18)	0.0605(10)	0.7019(18)	0.079(7)	0.0793(12)	0.0087(9)
	12,303	49,215	0.00200(24)	0.01100(42)	0.1164(13)	0.6455(34)	0.154(13)	0.1528(16)	0.0087(6)
	18,455	43,063	0.00292(21)	0.0100(7)	0.1760(22)	0.5855(36)	0.227(6)	0.2311(31)	0.00873(40)
	36,910	24,608	0.0064(6)	0.0062(5)			0.508(6)		0.0086(8)
	49,214	12,304	0.0093(7)	0.00310(44)			0.750(26)		0.0085(6)
1.25	8920	80,285	0.000093(22)	0.01136(45)	0.0878(35)	0.6946(36)	0.0081(19)	0.1122(44)	0.00774(33)
	12,303	49,215	0.00038(6)	0.00871(21)	0.1729(22)	0.6228(20)	0.041(6)	0.2172(21)	0.0063(7)
	24,607	36,911	0.00142(18)	0.00438(19)	0.3161(25)	0.4909(46)	0.245(25)	0.3917(34)	0.00407(38)
	36,910	24,608	0.00327(30)	0.00197(38)	0.4464(41)	0.3632(27)	0.624(39)	0.5514(41)	0.00376(38)
	49,214	12,304	0.0065(8)	0.00055(7)			0.922(11)		0.00506(33)

a Number in parentheses indicates the statistical uncertainty in the last decimal digit.

**Table 2:** Simulation Results: Vapor Phase Mole Fraction of Component A *x*_A_^″^, Surface Excess of Component A (Γ_A_^″^) and B (Γ_B_^″^), Vapor Phase Adsorbate Layer Mole Fraction of Component A *x*_A_^″,ads^, Layer Thickness of Component A (δ_A_^″^) and B (δ_B_^″^), and Contact Angle θ^a^

ξ_AB_	*x*_A_^″^/mol mol^–1^	Γ_A_^″^/σ^–2^	Γ_B_^″^/σ^–2^	*x*_A_^″,ads^/mol mol^–1^	δ_A_^″^/σ	δ_B_^″^/σ	θ/deg
0.70	0.2120(47)	0.222(20)	0.048(16)	0.816(48)	2.76(44)	2.9(8)	90.4(24)
	0.251(8)	0.425(18)	0.073(20)	0.850(34)	3.4(9)	3.3(11)	83.3(20)
	0.381(10)	1.004(37)	0.146(16)	0.871(13)	4.20(46)	4.32(18)	74.2(38)
	0.444(11)	1.645(29)	0.209(24)	0.883(10)	5.40(46)	5.5(7)	72(6)
	0.539(9)	2.43(6)	0.142(28)	0.942(9)	6.6(7)	6.6(5)	0
1.00	0.079(7)	0.0315(38)	0.026(14)	0.54(10)	2.07(44)	2.2(8)	83.6(46)
	0.154(13)	0.081(9)	0.037(15)	0.68(7)	2.40(46)	2.5(5)	59.3(22)
	0.227(6)	0.159(22)	0.051(15)	0.754(41)	2.8(9)	2.7(5)	38.4(12)
	0.508(6)	2.038(46)	1.255(31)	0.6188(21)	7.7(6)	7.6(5)	0
	0.750(26)	2.676(45)	0.626(23)	0.8103(42)	7.47(18)	7.6(6)	0
1.25	0.0081(19)	0.0026(15)	0.0166(45)	0.13(9)	1.8(8)	1.77(18)	103(5)
	0.041(6)	0.0111(27)	0.014(11)	0.42(12)	2.0(5)	1.92(44)	86.3(24)
	0.245(25)	0.045(7)	0.0110(38)	0.80(5)	2.1(8)	2.1(5)	60.4(18)
	0.624(39)	0.202(31)	0.019(6)	0.915(17)	2.7(5)	2.58(36)	32.2(9)
	0.922(11)	2.835(42)	0.779(8)	0.7844(36)	7.7(7)	8.0(7)	0

a Number in parentheses indicates the statistical uncertainty in the last decimal digit.

The composition of the fluid was varied by varying the overall ratio of particles of component A *N*_A_ and component B *N*_B_ in the simulation volume. Here, the total number of fluid particles was chosen such that a sufficient number of particles of both components was available to obtain acceptable statistics, cf. refs (30,31). For characterizing the fluid composition, we could have used the overall particle number fraction *N*_A_/(*N*_A_ + *N*_B_), that is, an average over all regions with fluid particles. Instead, we prefer using the mole fraction *x*_A_^″^ in the bulk vapor phase, which is easier to interpret. It gives a more direct information on the studied scenario and is independent of the simulation box size.

The simulation results are discussed in the following subsections.

### Bulk Phases

**Figure 5:**
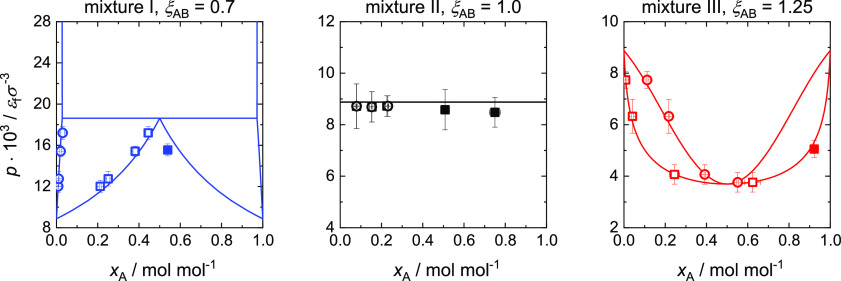
Phase diagrams calculated with the PeTS EOS for mixtures (lines) and bulk properties of the vapor phase (squares) and liquid phase (circles) of the simulations. The data of simulations with total wetting are depicted by full symbols. For these simulations, only the vapor data were calculated. Left: ξ_AB_ = 0.7 (blue); middle: ξ_AB_ = 1.0 (black); right: ξ_AB_ = 1.25 (red).

The bulk vapor phase and liquid phase properties obtained from the simulations for the cases with partial and total wetting were compared to phase diagrams calculated with the perturbed truncated and shifted (PeTS) equation of state (EOS). The PeTS EOS for the LJTS fluid with a cutoff radius *r*_c_ = 2.5σ was introduced first by Heier et al.^54^ for pure fluids and extended to binary mixtures by Stephan et al.^36^ It shows a good agreement with molecular simulation results for pure fluids and for mixtures.^36,48,49,54,55,60,61^Figure 5 shows the phase diagrams for the mixtures I, II, and III calculated with the PeTS EOS for mixtures together with the simulation results from the present work. For the simulations with total wetting (full symbols), only the bulk properties of the vapor phase (squares) are shown because no liquid droplet exists. For simulations with partial wetting (open symbols), the bulk properties of the vapor (squares) and liquid (circle) phase are depicted. The PeTS EOS calculates phase diagrams without any influence of interfaces. However, the liquid droplet in our simulations has a curved interface, which leads to an increase of the pressure inside the droplet. As the pressure inside the droplet was not measured, the total pressure of the vapor phase was also used for plotting the liquid phase results in Figure 5.

The vapor–liquid equilibrium (VLE) of the ideal mixture (mixture II) with ξ_AB_ = 1.0 (Figure 5, middle) calculated with the PeTS EOS for mixtures is a straight line because the fluids of the mixture behave like a pure fluid; the only difference between both fluid components is the different solid–fluid interaction. The mole fraction in the vapor and the liquid phase is the same and the vapor pressure is constant for all mole fractions. These characteristics are in good agreement with the bulk properties of the simulations, which show no influence of the solid wall on the bulk values; cf. Figure 5, middle. The pressure of all simulations is slightly smaller than that calculated with the PeTS EOS; however, these deviations are within the error bars. The symbols for the vapor and the liquid phase for the simulations with partial wetting lie on top of each other, that is, *x*_A_^″^ = *x*_A_^′^.

For mixture I with ξ_AB_ = 0.7 (Figure 5, left) a VLLE with a miscibility gap is observed. It can be seen that the bulk properties of the simulations with partial wetting are in good agreement with the calculations of the PeTS EOS for mixtures. The slight deviation for the data point corresponding to a simulation with total wetting (full symbol Figure 5, left) is not astonishing as there is no bulk liquid phase in this case.

The results for mixture III (with ξ_AB_ = 1.25) are shown on the right side of Figure 5. For this mixture, a high-boiling azeotrope is observed and the bulk properties of all simulations, even the one with total wetting, are in good agreement with the PeTS EOS calculations.

Figure 5 shows that the bulk phases of the simulations are in good agreement with the phase behavior calculated with the PeTS EOS.

### Contact Angle

**Figure 6:**
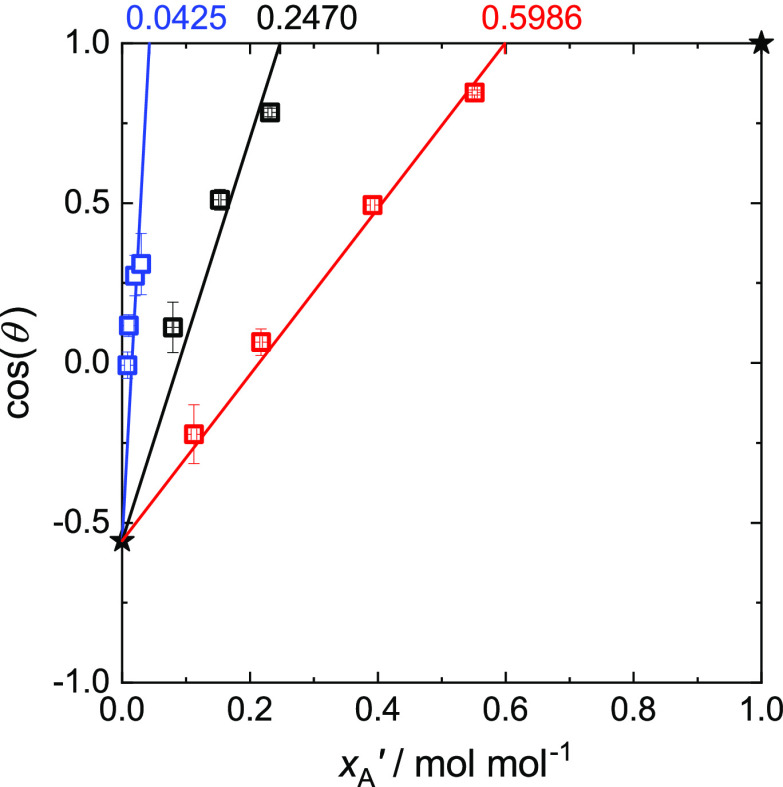
Contact angles θ observed in the present simulations (cf. Table 2) as a function of the liquid phase mole fraction of component A *x*_A_^′^ for the mixtures I (ξ_AB_ = 0.7; blue), II (ξ_AB_ = 1.0; black), and III (ξ_AB_ = 1.25; red). Symbols represent simulation results; lines represent the correlation given in the Supporting Information. The stars show the data for the pure fluids A and B.

The contact angle results obtained in the simulations of the present work are summarized in Figure 6. The cosine of the measured contact angle θ is plotted as function of the bulk liquid phase mole fraction of component A. Only results for simulations with partial wetting are shown. Besides the results for the three studied mixtures, the results for the two pure components A and B (θ_A_ = 0° and θ_B_ = 123.8°) are also shown (stars). The pure component contact angle values were determined with a correlation from Becker et al.^30^ The straight lines shown in Figure 6 are empirical correlations, as described in the Supporting Information.

Remarkably simple trends were found. For all studied mixtures, cos(θ) increases linearly with *x*_A_^′^, starting from the value for pure component B, that is, *x*_A_^′^ = 0 mol mol^–1^. The slopes differ for the different mixtures, leading to different *x*_A_^′^, for which cos(θ) becomes 1 (total wetting). For cos(θ) = 1, the empirical correlation leads to mixture I: *x*_A_^′^ = 0.0425 mol mol^–1^, mixture II: *x*_A_^′^ = 0.2470 mol mol^–1^, and mixture III: *x*_A_^′^ = 0.5986 mol mol^–1^. The strongest influence of increasing *x*_A_^′^ on the contact angle is observed for mixture I (ξ_AB_ = 0.7), in which the unlike fluid–fluid interactions are unfavorable; the weakest influence is observed for mixture III (ξ_AB_ = 1.25) with favorable unlike fluid–fluid interactions. This can be understood as a consequence of the stronger depletion of component B in the adsorbate layer, which leads to a stronger shielding of component B of the solid, for the unfavorable unlike fluid–fluid interactions. The depletion of component B is discussed in more detail in the following sections.

### Density Fields and Density Profiles

#### Mixture II (ξ_AB_ = 1.0)

**Figure 7:**
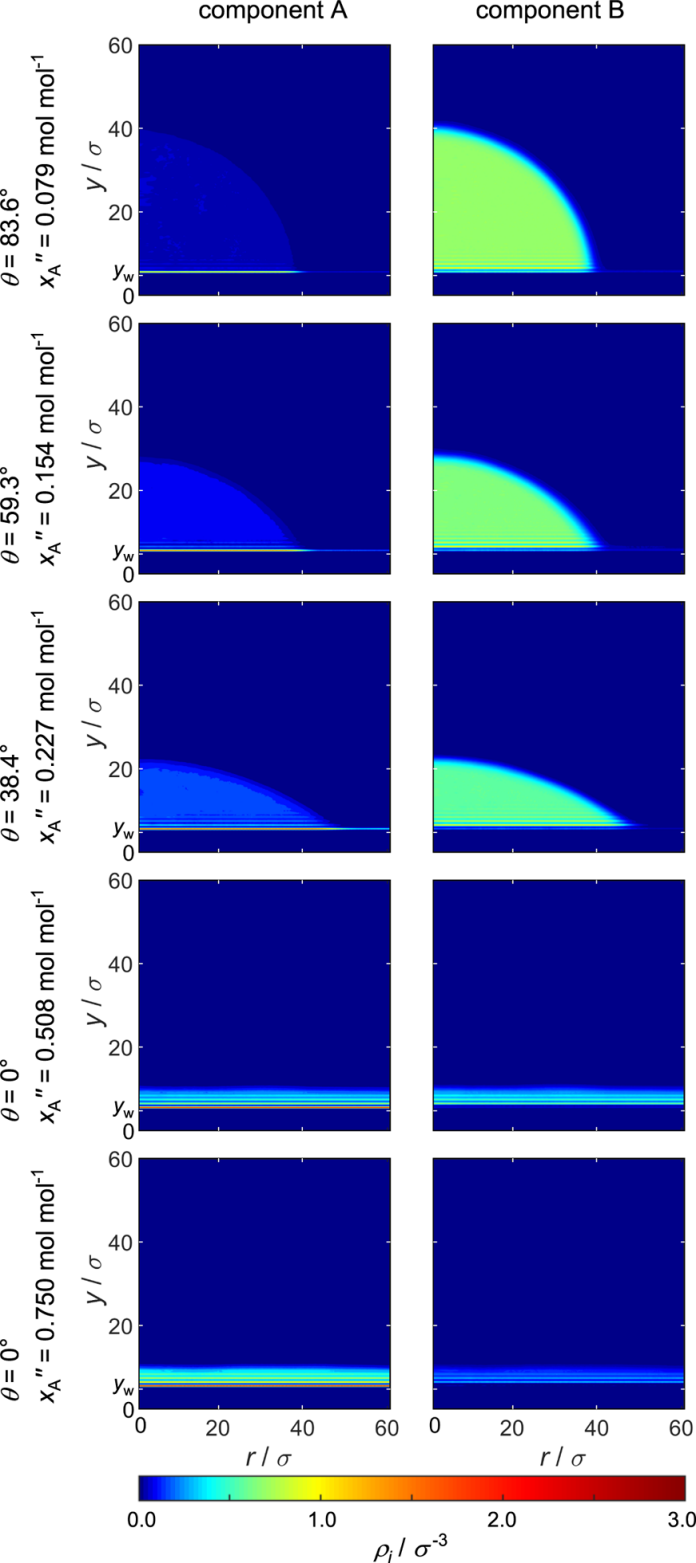
Density fields ρ_A_(*y*,*r*) (left) and ρ_B_(*y*,*r*) (right) for ξ_AB_ = 1.0. The center of the uppermost wall particles is indicated by *y*_w_ = 4.75σ.

**Figure 8:**
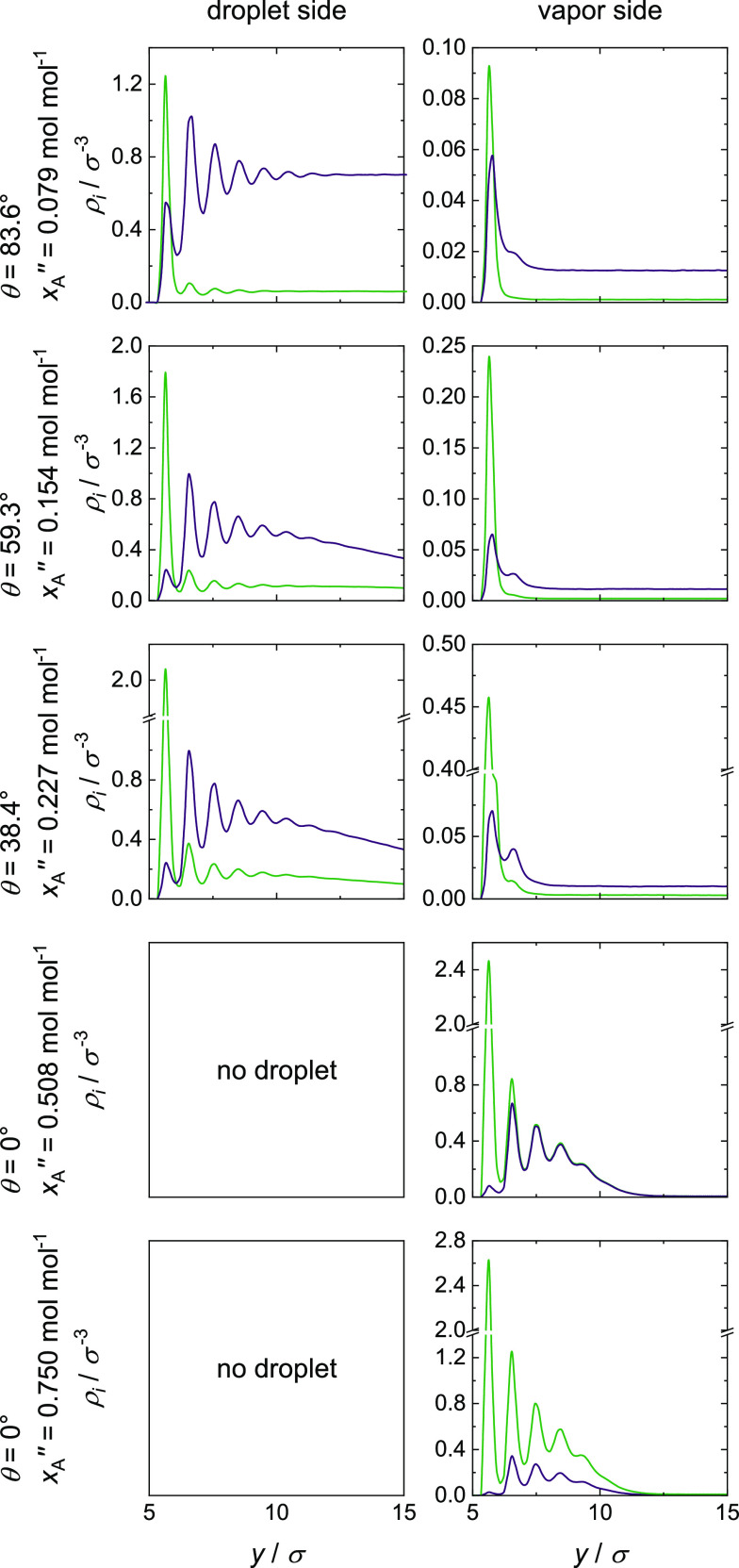
Density profiles ρ_A_(*y*) (green) and ρ_B_(*y*) (violet) on the droplet (left) and on the vapor side (right) for ξ_AB_ = 1.0.

Density fields ρ_*i*_(*y*,*r*) obtained from the simulations with the ideal mixture (mixture II, ξ_AB_ = 1.0) are shown in Figure 7; the corresponding density profiles ρ_*i*_(*y*) are shown in Figure 8 (note that the scale of the density axis in Figure 8 varies: the results for the vapor density profiles in the cases with partial wetting appear magnified). For mixture II, the only difference between the components A and B is the different strength of the solid–fluid interaction. Depending on the composition of the fluid, different wetting cases are observed: for high concentrations of component B, there is partial wetting, while for high concentrations of component A, there is total wetting. This behavior is caused by the pure component contact angles, that is, θ_A_ = 0° and θ_B_ = 123.8°.

The inspection of the three partial wetting simulations in Figure 7 reveals differences between the liquid phase and vapor phase adsorbate layer. Below the liquid droplet, a strong structuring of the fluid is visible close to the wall’s surface. For the two simulations with total wetting, a strong structuring of the fluid at the surface is observed as well. Comparing the partial density of component A (left side in Figure 7) and B (right side in Figure 7), a higher affinity of component A to the wall is observed; this is caused by ξ_sA_ > ξ_sB_.

The corresponding density profiles ρ_A_(*y*) and ρ_B_(*y*) (cf. Figure 8) give additional insights into the adsorbate layer: for partial wetting on the droplet side, a strong layering with up to seven density maxima is observed before the density levels out to the liquid bulk density. On the vapor side, only one or two maxima can be seen. This thin-film adsorption is a result of the much weaker vapor phase adsorption and accompanies with a small adsorbate layer thickness, which can be determined from the density profiles. For total wetting, a strong structuring with up to six maxima, that is, thick-film adsorption and a large adsorbate layer thickness, is observed. The results for the vapor phase adsorbate layer gathered here show a discontinuous transition from thin-film to thick-film adsorption. This transition takes place simultaneously with the transition from partial to total wetting.

Furthermore, the strong preference of component A to the solid leads to high concentrations of component A in the first adsorbate layer at the surface and to a depletion of component B in this layer, which increases with increasing *x*_A_^″^. This effect levels out with increasing distance from the wall’s surface; see, for example, the case with *x*_A_^″^ = 0.508 mol mol^–1^ for total wetting. From the third layer, that is, a distance larger than the cutoff radius of 2.5σ from the surface, no direct influence of the wall is present and the density profiles of component A and B are identical. The composition from the third layer of the vapor phase adsorbate layer is the same for both components and is determined by the fluid–fluid interaction. For partial wetting and a distance between the droplet and the wall’s surface smaller than the cutoff radius, the droplet is influenced by the solid wall as well as by the underlying adsorbate layer. For a distance larger than the cutoff radius, however, the droplet is only influenced by the adsorbate layer and not directly by the solid wall. The present simulations with mixture II show a distance between the droplet and the wall’s surface smaller than the cutoff radius and as a consequence, the droplet is influenced by both the wall and the underlying adsorbate layer. The distance of 2.5σ corresponds to 0.85 nm for argon with a size parameter of σ_Ar_ = 0.33916 nm.^32^ These findings are in good agreement with experimental data, which do not show any influence of the substrate on the wetting for adsorbate layers thicker than 1 – 2 nm; cf. ref (2).

#### Mixture I (ξ_AB_ = 0.7)

**Figure 9:**
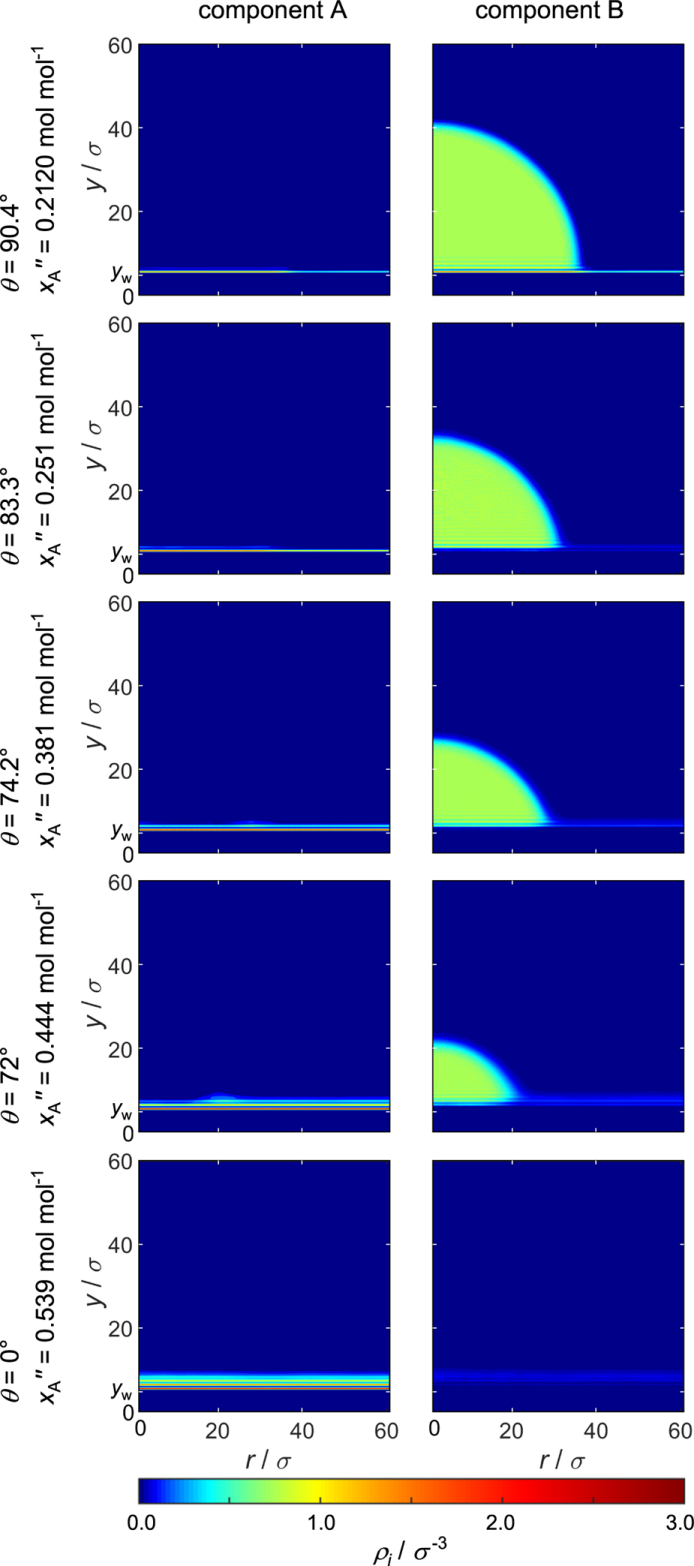
Density fields ρ_A_(*y*,*r*) (left) and ρ_B_(*y*,*r*) (right) for ξ_AB_ = 0.7. The center of the uppermost wall particles is indicated by *y*_w_ = 4.75σ.

**Figure 10:**
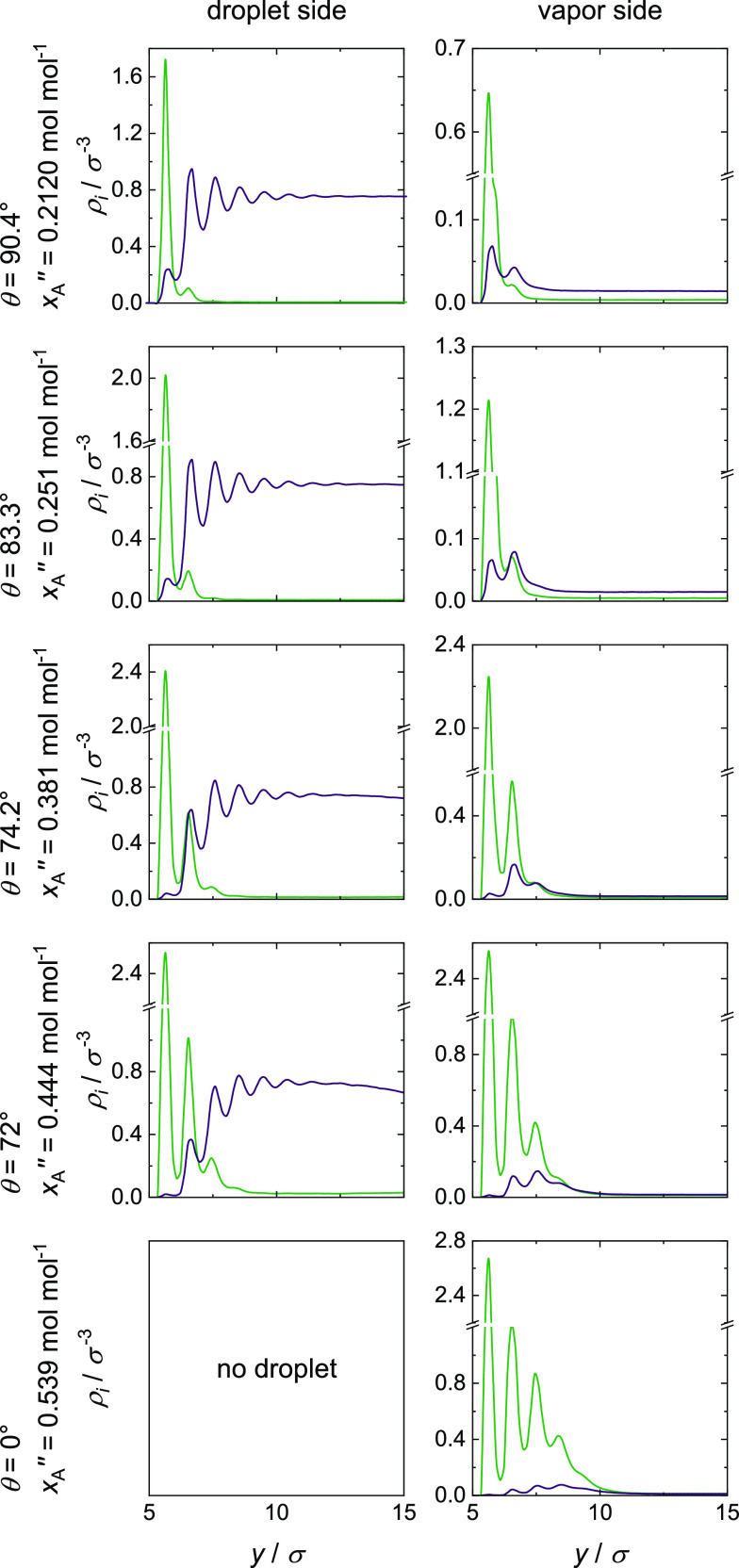
Density profiles ρ_A_(*y*) (green) and ρ_B_(*y*) (violet) on the droplet (left) and on the vapor side (right) for ξ_AB_ = 0.7.

The simulations with the hetero-azeotropic mixture I (ξ_AB_ = 0.7) lead to the density fields ρ_*i*_(*y*,*r*) shown in Figure 9 and the corresponding density profiles ρ_*i*_(*y*) shown in Figure 10. Again, component A has a higher affinity to the surface than component B, but in addition, the unlike fluid–fluid interaction is unfavorable. Therefore, the phenomenology of the observed wetting behavior differs significantly from that observed for the ideal mixture II. In the cases with partial wetting, which are observed for low *x*_A_^″^, basically a drop of component B is sitting on an adsorbate layer, which is rich in component A; cf. Figure 9. By increasing *x*_A_^″^, the contact angle decreases and, beyond a certain point, total wetting is observed.

The corresponding density profiles ρ_*i*_(*y*) (cf. Figure 10) show the same behavior as already seen for mixture II: the first adsorbate layer is rich in component A, whereas component B is depleted. However, the concentration of component B in this first layer is even lower than in corresponding cases for mixture II. This results from the unfavorable unlike fluid–fluid interaction for this mixture. With increasing *x*_A_^″^, the thickness of the adsorbate layer underneath the droplet, which is rich in component A, increases, and therefore, the direct influence of the solid wall on the droplet decreases. Due to the fact that the droplet, which is rich in component B, sits basically on this adsorbate layer, the increase in the adsorbate layer thickness leads to an increase of the distance from the wall’s surface to the droplet. For all simulations with partial wetting, except the simulation with *x*_A_^″^ = 0.444 mol mol^–1^, the distance between the droplet and the surface is smaller than the cutoff radius and therefore, the droplet is influenced by the adsorbate layer as well as by the solid wall. For the simulation with *x*_A_^″^ = 0.444 mol mol^–1^, the droplet is only influenced by the underlying adsorbate layer and the direct influence of the solid wall vanishes. The underlying adsorbate layer leads to a contact angle of θ = 72°.

The vapor phase adsorbate layer shows a continuous transition from thin-film to thick-film adsorption, that is, the adsorbate layer thickness increases steadily with increasing *x*_A_^″^. This transition does not take place simultaneously with the transition from partial wetting to total wetting, as observed for mixture II.

#### Mixture III (ξ_AB_ = 1.25)

**Figure 11:**
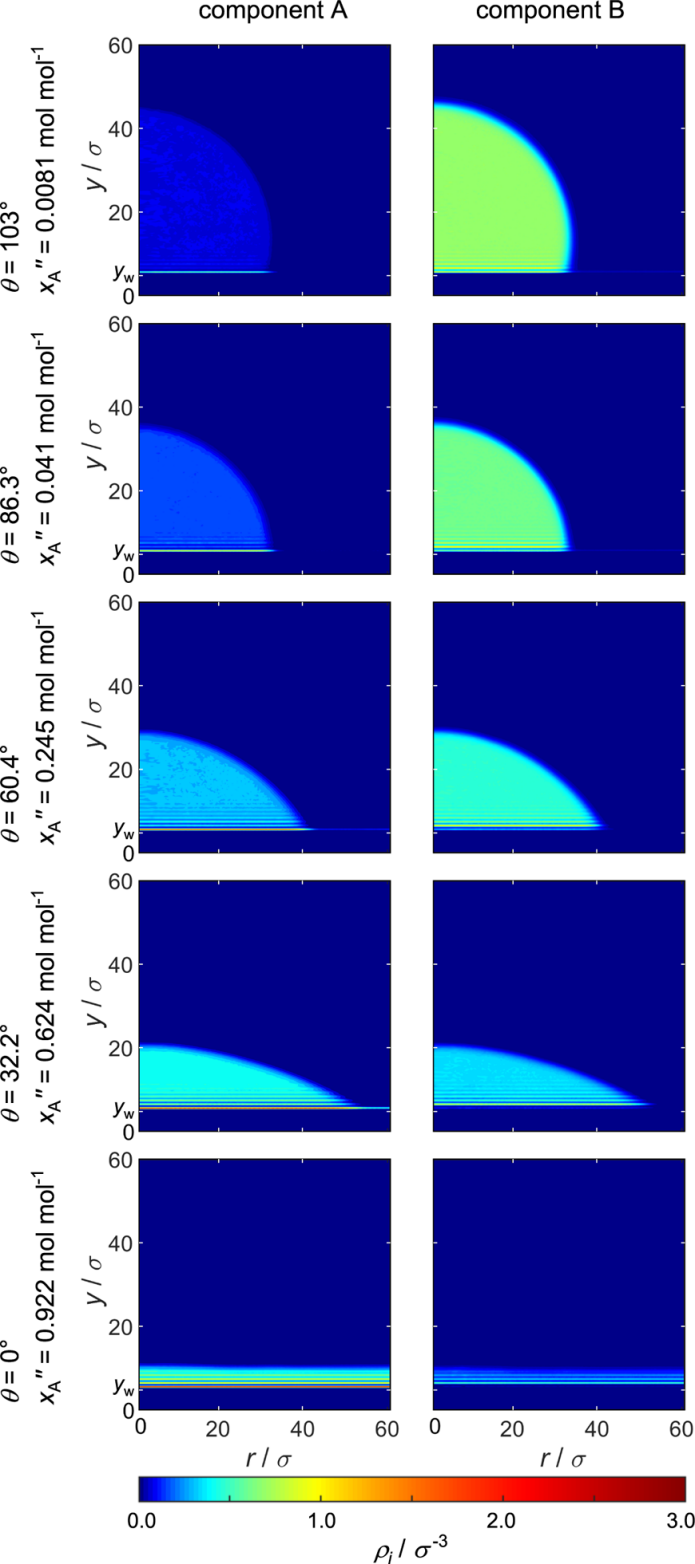
Density fields ρ_A_(*y*,*r*) (left) and ρ_B_(*y*,*r*) (right) for ξ_AB_ = 1.25. The center of the uppermost wall particles is indicated by *y*_w_ = 4.75σ.

**Figure 12:**
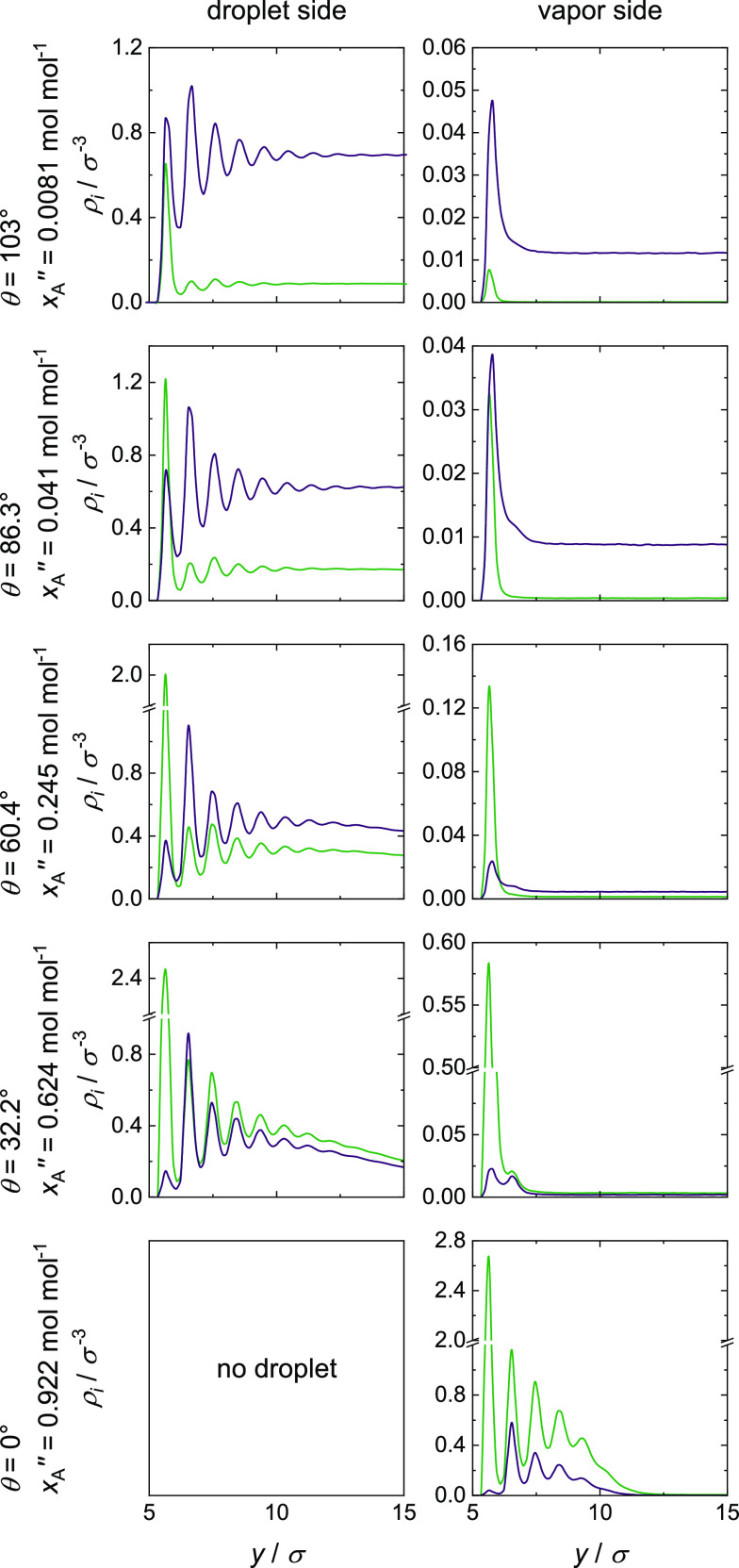
Density profiles ρ_A_(*y*) (green) and ρ_B_(*y*) (violet) on the droplet (left) and on the vapor side (right) for ξ_AB_ = 1.25.

The density fields ρ_*i*_(*y*,*r*) obtained from the simulations with mixture III (ξ_AB_ = 1.25), which forms a low-boiling azeotrope, are shown in Figure 11 and the corresponding density profiles ρ_*i*_(*y*) are shown in Figure 12. The preferential adsorption of component A at the wall’s surface and the layering structure of the adsorbate layer also appear for mixture III. However, the high affinity of both fluid components in this mixture leads to an increased homogeneity of the fluid compared to mixture I and II. Again, by increasing *x*_A_^″^, the contact angle decreases and total wetting is observed. For this mixture, total wetting is observed for the highest value of *x*_A_^″^. This results from the phase behavior of the high-boiling azeotrope, where for *x*_A_^″^ > 0.5 mol mol^–1^ it is *x*_A_^′^ < *x*_A_^″^ and therefore, component A has less influence on the droplet than for the other mixtures with same *x*_A_^″^.

The corresponding density profiles ρ_*i*_(*y*) shown in Figure 12 show the same behavior as seen for mixtures I and II; however, there is a difference: for low *x*_A_^″^, component B is richer in the first adsorbate layer than component A. This results not only from the small *x*_A_^″^ but also from the favorable unlike fluid–fluid interaction. For higher *x*_A_^″^, it can be seen that the first adsorbate layer is still rich in component A and component B is still depleted, however, not as much as for mixture I and II. For this mixture, the distance between the droplet and the surface is smaller than the cutoff radius and as a consequence, the droplet is influenced by both the solid wall and the adsorbate layer underneath the droplet. This is similar to most simulations in the present work except for one simulation with mixture I. The influence of the wall on the droplet decreases with increasing *x*_A_^″^.

As already observed for mixture II for the vapor phase adsorbate layer, a discontinuous transition from thin-film to thick-film is observed. This change in the adsorbate layer thickness takes place simultaneously with the transition from partial to total wetting.

### Adsorption Isotherms

**Figure 13:**
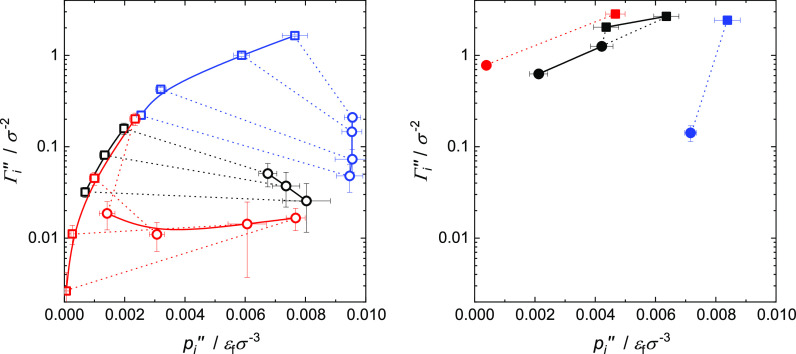
Vapor phase adsorption isotherms of component A (squares) and B (circles) versus the partial pressure of each fluid component *p*_*i*_^″^ for ξ_AB_ = 0.7 (blue), 1.0 (black), and 1.25 (red). The solid lines are a guide to the eye. The results that belong to the same simulation are connected by dotted lines. Left: results of simulations with partial wetting (open symbols); right: results of simulations with total wetting (full symbols).

The vapor phase adsorption data for the cases with partial and total wetting were used to determine adsorption isotherms. The adsorption isotherms for both fluid components obtained from the results for the three mixtures studied here are shown in Figure 13. The surface excess Γ_*i*_^″^ (cf. eq 7 in the Appendix) describes the number of particles of a component on the solid wall per area and is plotted as a function of the partial pressure. The vapor phase in the present simulations was almost ideal; therefore, the partial pressures *p*_A_^″^ and *p*_B_^″^ in the vapor phase can be defined as *p*_A_^″^ = *x*_A_^″^·*p*″ and *p*_B_^″^ = (1 – *x*_A_^″^)*p*″. In the diagram on the left side of Figure 13, the results from the simulations with partial wetting are shown and on the right side the results from simulations with total wetting. A logarithmic scale is used to improve the representation of the surface excess for small surface excess values. The surface excess results for the component A and B from the same simulation are connected by dotted lines. The adsorption of component A on the surface is always much stronger than that of component B (for the same partial pressure, the isotherms of component A lie far above that of component B in all cases).

We start with the discussion of the results for partial wetting (Figure 13, left). The results for component A are as expected: the surface excess increases steadily with increasing partial pressure. Interestingly, the results for the three mixtures lie basically on the same line, that is, there is no influence of the strength of the unlike fluid–fluid interactions. This means that the surface excess of fluid component A is not affected by fluid component B.

This is, however, not the case vice versa: the adsorption isotherms of component B for the three studied mixtures differ strongly and astonishing trends are observed for mixtures II and III. The adsorption isotherms of component B for mixture I (blue) are as expected: with increasing *p*_B_^″^, the surface excess of component B increases. For mixtures II and III, a different behavior is observed: with increasing surface excess of component A, the ideal or favorable fluid–fluid interaction leads to an increasing surface excess of component B, even though *p*_B_^″^ is decreasing; component B is attracted by component A. For most simulations conducted in the present work, the amount of component A in the first adsorbate layer was much larger than that of component B. For these cases, the influence of the solid on component B is shielded by component A. However, for the simulations with *x*_A_^″^ = 0.0081 and 0.041 mol mol^–1^ of mixture III (largest *p*_B_^″^ in Figure 13), the shielding is reduced due to the small amount of component A in the first adsorbate layer (cf. Figure 12) and the solid’s influence on component B is increased. This results in a higher surface excess than with shielding.

The information obtained for the adsorption isotherms for the total wetting case is patchy (cf. Figure 13), but as far as trends can be observed, they are in line with expectations: the surface excess increases with increasing partial pressure (mixture II) and the surface excess of component B is smaller than that of component A. Furthermore, it can be seen that the surface excess for mixtures II and III increases drastically compared to the simulations with partial wetting, both for component A and B. This behavior characterizes the transition from thin-film to thick-film adsorption together with the transition from partial to total wetting. For mixture I, however, the continuous change from thin-film to thick-film adsorption for fluid component A, which was already seen in Figure 10, is observed.

In the present work, results for the adsorbate layer thickness δ_*i*_^″^ of each component were also obtained. The layer thickness shows the same behavior as the adsorption isotherms of component A and δ_A_ and δ_B_ are the same. The results of the adsorbate layer thickness are presented in the Supporting Information.

**Figure 14:**
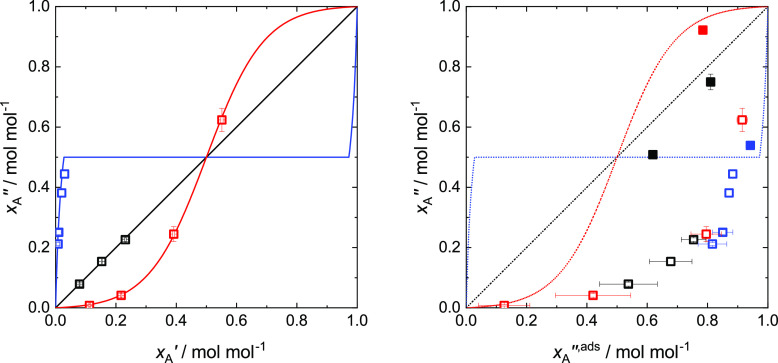
Vapor phase mole fraction *x*_A_^″^, liquid phase mole fraction *x*_A_^′^, and vapor phase adsorbate layer mole fraction *x*_A_^″,ads^ for ξ_AB_ = 0.7 (blue), 1.0 (black), and 1.25 (red) calculated with the PeTS EOS for mixtures (lines) and determined by the simulations (symbols) for partial wetting (open symbols) and total wetting (full symbols). Left: *x*_A_^″^ as function of *x*_A_^′^; right: *x*_A_^″^ as function of *x*_A_^″,ads^ (simulations). *x*_A_^″^ as function of *x*_A_^′^ calculated with the PeTS EOS for mixtures is depicted for comparison (dotted lines).

In Figure 14 two McCabe–Thiele diagrams are shown (plots of vapor phase mole fraction over the liquid phase mole fraction for the studied temperature). On the left side of Figure 14, the McCabe–Thiele diagram for the three mixtures as determined with the PeTS EOS (lines) is shown, that is, it gives information on the bulk properties. The differences between the three mixtures become evident: mixture II is ideal and as the two pure components are the same, there is no difference between the composition of the two phases. Mixture I is hetero-azeotropic and mixture III has a high-boiling azeotrope. Both mixtures show curves that are symmetric due to the identity of the two pure components. The results for the bulk properties determined in the simulations with partial wetting are shown as symbols. They agree perfectly with the lines determined with the PeTS EOS.

In the McCabe–Thiele diagram on the right side of Figure 14, the liquid phase mole fraction is that of the vapor phase adsorbate layer. The results determined with the PeTS EOS are the same as on the left side and are only indicated as dotted lines to facilitate the comparison with the simulation results (symbols). For partial wetting (open symbols), compared to the VLE, component A is enriched strongly in the vapor phase adsorbate layer, which is a consequence of the strong attraction of the wall for component A. This observation is expected; however, also an unexpected behavior is observed: all partial wetting simulation results (open symbols) lie basically on one curve independent of the mixture, even though strong differences were observed in the adsorption isotherms (cf. Figure 13) and also in the structure of the adsorbate layer (cf. Figure 8, 10, and 12). This means that the mole fraction of the vapor phase adsorbate layer can be predicted from that of the vapor phase without taking into account the strength of the unlike fluid–fluid interactions. It is determined only by the solid–fluid interactions ξ_sA_ and ξ_sB_.

The results obtained from the simulations with total wetting (full symbols) are different than those obtained for the simulations with partial wetting. They lie in the vicinity of the corresponding bulk values determined by the PeTS EOS and show a decreased separation of component A between the bulk vapor and the adsorbate. This results from the thick-film adsorbate layer appearing in the simulations with total wetting. The thick-film adsorbate layer leads, due to its thickness, to an increased influence of the fluid–fluid interaction and to a decreased influence of the solid–fluid interaction (only the first two adsorbate layers are influenced directly by the solid wall; the thick-film adsorbate layer, however, shows up to six adsorbate layers, cf. Figure 8, 10, and 12) on the adsorbate layer. Due to the small influence of the solid wall on the full thick-film adsorbate layer, the composition is more liquid-like. The total wetting result for mixture I (blue full symbol) happens to lie on the curve obtained from the results for partial wetting; however, this seems to be a coincidence.

Concentration profiles of the vapor phase adsorbate layer for the simulations with total wetting give a more detailed insight into the composition of the adsorbate layer and can be found in the Supporting Information.

### Three-Phase Contact

**Figure 15:**
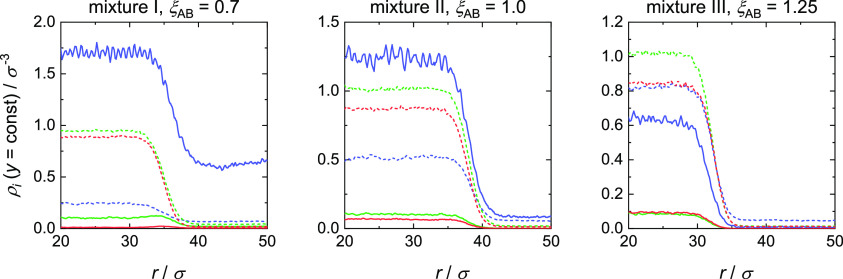
Fluid component density profiles ρ_*i*_(*y* = const, *r*) of component A (solid line) and B (dotted line) for *y* = 5.633σ (blue), *y* = 6.534σ (green), and *y* = 7.436σ (red). Left: mixture I (ξ_AB_ = 0.7) for *x*_A_^″^ = 0.2120 mol mol^–1^; middle: mixture II (ξ_AB_ = 1.0) for *x*_A_^″^ = 0.079 mol mol^–1^; right: mixture III (ξ_AB_ = 1.25) for *x*_A_^″^ = 0.0081 mol mol^–1^.

The three-phase contact separates the adsorbate layer into a vapor phase adsorbate layer and a liquid phase adsorbate layer. The transition from the liquid to the vapor phase adsorbate layer at the three-phase contact is shown in detail in Figure 15 for one simulation of each mixture (left: mixture I, ξ_AB_ = 0.7, *x*_A_^″^ = 0.2120 mol mol^–1^; middle: mixture II, ξ_AB_ = 1.0, *x*_A_^″^ = 0.079 mol mol^–1^; right: mixture III, ξ_AB_ = 1.25, *x*_A_^″^ = 0.0081 mol mol^–1^). Therefore, density profiles at a constant *y*-value ρ_*i*_(*y* = const, *r*) are plotted as function of *r*. Here, these profiles are shown for the first three density maxima of the adsorbate layers, that is, at *y* = 5.663, 6.534, and 7.436σ. As expected, the density on the droplet side is always larger than that on the vapor side for both components. For *y* = 6.534σ (green) and *y* = 7.436σ (red), the transition from the liquid to the vapor side for each mixture is rather similar to the vapor–liquid interface observed for these mixtures for planar interfaces. For mixture I, an enrichment of component A at the interface is observed, whereas for mixtures II and III, no enrichment is observed. This is in good agreement with the findings of Stephan et al.,^36,60,61^ who investigated the vapor–liquid interfaces of binary LJTS fluid mixtures with density gradient theory and MD simulations. They found an enrichment for low-boiling azeotropic mixtures and no enrichment for high-boiling azeotropic mixtures and a quasi-ideal mixture. In contrast to the two upper layers, for mixture I in the three-phase contact, that is, *y* = 5.663σ (blue), no enrichment of component A at the interface is observed. This layer close to the wall is strongly influenced by the solid–fluid interaction such that the vapor–liquid interface is superimposed in the three-phase contact by the solid–fluid interaction. This superimposing decreases with increasing distance from the wall’s surface. For mixtures II and III for *y* = 5.663σ (blue), a strong influence of the solid–fluid interaction is also observed. However, the interfacial behavior stays the same.

## Conclusions

In the present study, the wetting of a planar wall with binary fluid mixtures was investigated with MD simulations. Three different mixtures were studied: while the pure components A and B were identical, the unlike fluid–fluid interaction was varied, resulting in a mixture with a (low-boiling) hetero-azeotrope, an ideal mixture, and a mixture with a high-boiling azeotrope. Furthermore, the composition of the binary mixtures was varied. Component A was attracted more strongly by the wall than component B. All interactions (i.e., fluid–fluid, solid–fluid, and solid–solid) were described by a LJTS potential with a cutoff radius of 2.5σ.

The simulation results can be classified into two cases: partial wetting (preferentially for high concentrations of component B) and total wetting (preferentially for high concentrations of component A). Remarkably simple results were obtained for the contact angle: starting from the contact angle for pure component B, the cosine of the contact angle increases linearly with the concentration of component A in the liquid phase for all studied mixtures. The decrease of the contact angle is stronger for the hetero-azeotropic mixture with unfavorable unlike fluid–fluid interactions than for the mixture with the high-boiling azeotrope with favorable unlike fluid–fluid interactions, that is, total wetting is reached for lower concentrations of A for the hetero-azeotropic mixture.

Due to the strong preference of component A, high concentrations of component A in the first adsorbate layer and a depletion of component B in that layer are observed. This effect levels out with increasing distance from the wall’s surface and vanishes at distances above 2.5σ from the surface. Then, only the fluid–fluid interactions determine the wetting. The strong adsorption of component A leads to a shielding of component B from the solid. Both, thin-film and thick-film adsorption from the vapor phase were observed. Total wetting always resulted in thick-film adsorption and partial wetting mostly in thin-film adsorption, except for the hetero-azeotropic mixture and high concentrations of component A. There, thick-film adsorption was observed. For this mixture, the transition from thin-film to thick-film took place continuously. For the other two mixtures, it took place in a discrete manner upon the transition from partial to total wetting.

The surface excess of component A shows no influence of the strength of the unlike fluid–fluid interactions; however, this is not the case for the surface excess of component B, which depends strongly on the unlike fluid–fluid interactions.

An unexpected behavior was observed in the McCabe–Thiele diagram relating the vapor phase adsorbate layer mole fraction to that in the bulk vapor phase: for partial wetting, all simulation results lie on one curve, independent of the mixture. This means that the mole fraction of the vapor phase adsorbate layer can be predicted from that of the vapor phase without taking into account the strength of the unlike fluid–fluid interactions; it is determined only by the solid–fluid interactions. For total wetting, the composition of the adsorbate layer is more liquid-like and the separation of component A is decreased compared to partial wetting.

In the three-phase contact, the behavior of the vapor–liquid interface is superimposed by the solid–fluid interaction. For an increasing distance from the solid, the corresponding vapor–liquid interface of each mixture was observed.

Molecular simulation studies enable systematic studies of the influence of molecular parameters on the adsorption and wetting of mixtures. Only a very simple scenario was investigated here: the attractive interactions were dispersive, the two pure fluids were identical, and only two molecular parameters were varied. Despite this, a wealth of phenomena was observed and could be explained. The approach can obviously be extended to many other interesting cases.

## Appendix

### Further Information Regarding the Data Evaluation

For partial wetting, the liquid droplet and the bulk liquid region are characterized by the contact angle θ, the droplet radius *R*_d_, the component density ρ_*i*_^′^ with *i* = A, B in the liquid phase, and the mole fraction *x*_A_^′^ in the liquid phase. These quantities are calculated using the fluid component density fields ρ_*i*_(*y*,*r*). For the calculation of the contact angle θ and the droplet radius *R*_d_, the total density ρ(*y*,*r*) = ρ_A_(*y*,*r*) + ρ_B_(*y*,*r*) was used. The vapor–liquid interface of the sessile drop was defined by the arithmetic mean density (ρ′ + ρ″)/2, where ρ′ and ρ″ are the bulk liquid and bulk vapor density. A circle was fitted to the result of the arithmetic mean density, neglecting points near the wall’s surface. The intersection of the circle with the wall’s surface defines the droplet radius *R*_d_. The contact angle θ of the sessile drop was calculated as the angle between the surface of the wall and the tangent to the circle at the intersection with the wall’s surface.

The bulk quantities of the liquid phase (ρ_*i*_^′^ and *x*_A_^′^) were determined by averaging all values inside the liquid droplet excluding the interfacial region between the liquid and the vapor phase by a distance of 5σ from the circle fit and by excluding the layering of the adsorbate layer underneath the liquid droplet. The liquid phase mole fraction of component A *x*_A_^′^ was calculated using eq 5.

5

The quantities of the bulk vapor phase and the vapor phase adsorbate layer were determined equally for partial wetting and total wetting. The quantities of the liquid phase adsorbate layer underneath the droplet were not calculated due to the reason stated in the main part of this article. The vapor phase is characterized by the component densities ρ_*i*_^″^, the mole fraction *x*_A_^″^, and the total pressure *p*″, whereas ρ_*i*_^″^ and *x*_A_^″^ were determined using the vapor side density profiles ρ_*i*_^v^(*y*) and *p*″ was determined using pressure profiles *p*(*y*). The bulk densities ρ_*i*_^″^ in the vapor phase were calculated by averaging the data of ρ_*i*_^v^(*y*) over all *y* that belong to the bulk vapor phase. These *y*-values were neither influenced by the layering of the adsorbate layer nor by the membrane. The uppermost value where the membrane did not influence the bulk data is *y*_1_. The vapor phase mole fraction of component A *x*_A_^″^ was determined in the same way as the liquid phase mole fraction of component A

6

The total pressure *p*″ in the vapor phase was calculated using pressure profiles *p*(*y*), which were averaged over all *r* for cases with partial wetting and total wetting. These pressure profiles were determined using the intermolecular virial based on the method of Irving and Kirkwood^64,65^ and it was one-third of the trace of the pressure tensor and was sampled with the same block size and number of bins as the fluid component density profiles. The pressure tensor has two distinct entries, that is, the tangent pressure and the normal pressure. Both differ strongly in the interfacial regions (adsorbate layer and vapor–liquid interface of the droplet). However, they are equal in the bulk phase, which is the only region we are interested in. The total pressure in the vapor phase was determined by averaging the data of *p*(*y*) over all *y*-values that belong to the bulk vapor phase. For total wetting, these *y*-values are all values that were not influenced by the layering of the adsorbate layer and were smaller than *y*_1_. For partial wetting, the corresponding *y*-values were smaller than *y*_1_ and greater than the height of the droplet. The total pressure in the liquid phase was not determined, as already stated in the main part of this article.

The vapor phase adsorbate layer is characterized by the surface excess Γ_*i*_^″^, the adsorbate layer mole fraction of component A *x*_A_^″,ads^, and the layer thickness δ_*i*_^″^ of the adsorbate layer. These quantities were determined using ρ_*i*_^v^(*y*). The surface excess Γ_*i*_^″^ was calculated using the following equation

7

with *y*_0_ and *y*_1_ as integration limits. For determining the surface excess, Gibbs dividing surface was set to the surface of the solid wall, which was at *y* = 5.25σ and therefore, the integration start was *y*_0_ = 5.25σ in eq 7; cf. Figure 4. This is in contrast to our previous study,^31^ where *y*_0_ was the first intersection of the fluid component density profile with the component density in the vapor phase. The end of integration was *y*_1_. The vapor phase adsorbate layer mole fraction of component A *x*_A_^″,ads^ is an average value of the mole fraction in the vapor phase adsorbate layer and was determined by

8

The adsorbate layer thickness was calculated using:

9

where *y*_*i*,e_ was the upper bound of the layer thickness. It was found by the intersection of ρ_*i*_^v^(*y*) with 1.15 ρ_*i*_^″^ and is depicted in Figure 4.

## Supporting Information Available

Fixation of the atomistic wall, the contact angle correlation, the layer thickness, and the composition in the adsorbate layer for total wetting (PDF)
Averaged fluid component density profiles ρ_i_(*y*) for all adsorption simulations (XLSX)
