# Supplementary material for: Molecular Dynamics Study of Wetting and Adsorption of Binary Mixtures of the Lennard-Jones Truncated and Shifted Fluid on a Planar Wall
Source: Langmuir. 2021 Jun 7;37(24):7405–19. doi: 10.1021/acs.langmuir.1c00780 (PMC8280723; doi:10.1021/acs.langmuir.1c00780)
Supplement: Supplementary file 1 — la1c00780_si_001.pdf [file la1c00780_si_001.pdf]

Molecular Dynamics Study of Wetting and Adsorption of Binary Mixtures of the Lennard-Jones Truncated and Shifted Fluid on a Planar Wall

Michaela Heier,† Simon Stephan,† Felix Diewald,‡ Ralf Müller,‡
Kai Langenbach,*,†,¶ and Hans Hasse†

†*Laboratory of Engineering Thermodynamics, Technische Universität Kaiserslautern, Kaiserslautern, Germany*

‡*Institute of Applied Mechanics, Technische Universität Kaiserslautern, Kaiserslautern, Germany*

¶*Thermal Separation Science (endowed professorship of the state Tyrol), University of Innsbruck, Austria*

E-mail: kai.langenbach@uibk.ac.at

Phone: +43 512 50755200

Supplementary Information

Fixation of the Atomistic Wall

The simulation scenario used in the present work contains an atomistic wall, which is fixed at the bottom of the simulation box by applying an external Lennard-Jones truncated and shifted (LJTS) 9-3 potential with a cutoff at $y_\mathrm{c} = 2.5\ \sigma$. The LJTS 9-3 potential is defined

as:

$$u_{\mathrm{LJ93}}(y) = \frac{4}{3}\pi\varepsilon_{\mathrm{s}}\rho_{\mathrm{s}}\sigma^3 \left[\frac{1}{15}\left(\frac{\sigma}{y}\right)^9 - \frac{1}{2}\left(\frac{\sigma}{y}\right)^3\right] \tag{S1}$$

$$u_{\mathrm{LJTS93}}(y) = \begin{cases} u_{\mathrm{LJ93}}(y) - u_{\mathrm{LJ93}}(y_{\mathrm{c}}) & y \leq y_{\mathrm{c}} \\ 0 & y > y_{\mathrm{c}} \end{cases} \tag{S2}$$

and is located at $y = 0\ \sigma$. The energy parameter ε_{s}, the size parameter σ, and the solid density ρ_{s} are the same as for the atomistic wall. Due to the cutoff $y_{\mathrm{c}} = 2.5\ \sigma$ and a height of the atomistic wall which is higher than 2.5 σ, the LJTS 9-3 potential is only interacting with the lower layers of the atomistic wall and not with the fluids.

Contact Angle Correlation

The contact angle data of the present work's simulations can be described by a linear empirical correlation:

$$\cos(\theta) = x'_{\mathrm{A}} \cdot c + \cos(\theta_{\mathrm{B}}) \tag{S3}$$

with $c = 36.6$, 6.3, and 2.6 for $\xi_{\mathrm{AB}} = 0.7$, 1.0, and 1.25, respectively. The correlation starts for all unlike fluid-fluid interactions at $\cos(\theta_{\mathrm{B}})$ for $x'_{\mathrm{A}} = 0.0$ mol mol^{-1} and increases for increasing x'_{A}. With this correlation the transition from partial wetting to total wetting can be estimated as well as the contact angles of the binary mixtures for different ξ_{AB} and varying x'_{A}. The transition from partial to total wetting takes place for $\cos(\theta) = 1.0$: it is $x'_{\mathrm{A}} \approx 0.0425$, 0.2470, and 0.5986 mol mol^{-1} for $\xi_{\mathrm{AB}} = 0.7$, 1.0, and 1.25, respectively.

Layer Thickness

The vapor phase adsorbate layer thickness is determined component wise for component A and B and is shown in Figure S1 for the simulations with partial wetting and total wetting. On the left side, results for simulations with partial wetting are depicted and on the right side for total wetting. The vapor phase adsorbate layer thickness δ''_i is basically the same

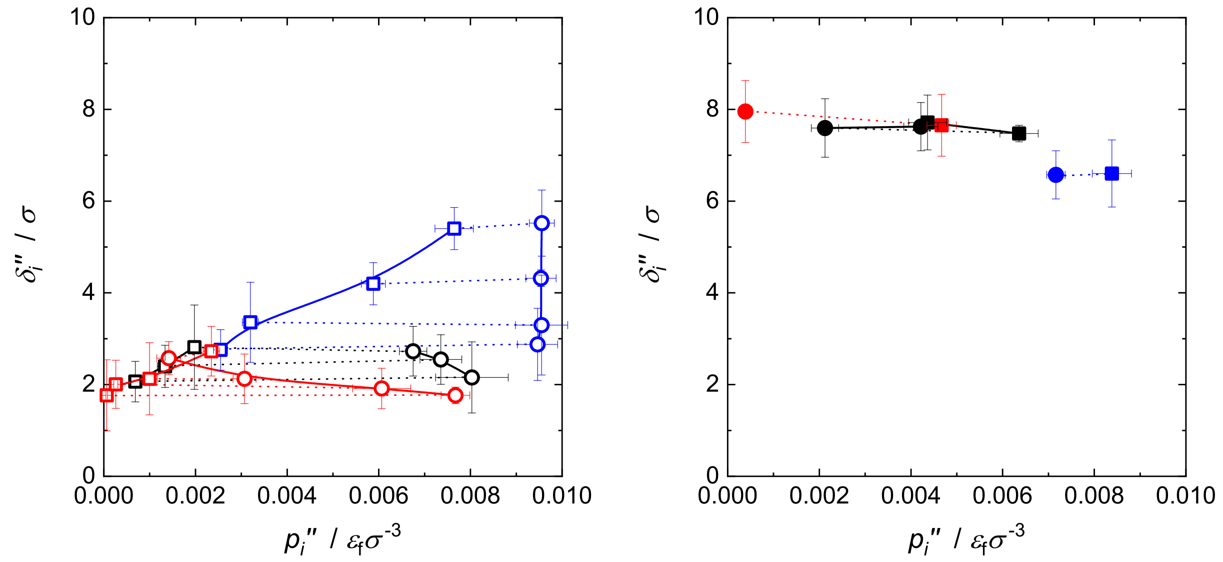

Figure S1: Layer thickness of component A (squares) and B (circles) versus the partial pressure of each fluid component p_i'' for $\xi_{AB} = 0.7$, (blue), 1.0 (black), 1.25 (red). The solid lines are a guide to the eye. The results that belong to the same simulation are connected by dotted lines. Left: results of simulations with partial wetting (open symbols); right: results of simulations with total wetting (full symbols).

for component A and B (dotted lines, which connect the results of component A and B of one simulation, are horizontal) for all three mixtures. Even for the unfavorable unlike fluid-fluid interaction, no difference in the adsorbate layer thickness is observed. Besides, the adsorbate layer thickness curves behave quite similar to the adsorption isotherms: the unlike fluid-fluid interactions do not influence δ_A'' whereas δ_B'' is influenced strongly. On the right side of Figure S1, for mixture II and III, the adsorbate layer thickness increases strongly with the transition from partial wetting to total wetting, i.e. the transition from thin-film to thick-film adsorption. For mixture I, this transition is continuous.

Composition in the Adsorbate Layer for Total Wetting

The results of the simulations with partial wetting show a high separation of component A by the solid wall, which is decreased for the simulations with total wetting. This change of

behavior results of the thick-film vapor phase adsorbate layer observed for the simulations with total wetting. Concentration profiles $x_{\mathrm{A}}^{\mathrm{v}}(y)$ in the adsorbate layer for simulations with total wetting give an additional insight into the adsorbate layer. They are shown in Figure S2 as well as the corresponding density profiles $\rho_i^{\mathrm{v}}(y)$ and the bulk liquid phase mole fraction of component A calculated with the PeTS EOS. The density profiles of component A (green) and B (violet) are depicted as a guide. For the ideal mixture II ($\xi_{\mathrm{AB}} = 1.0$, right top and left bottom) a strong influence of the solid-fluid interaction for distances smaller than 2.5 σ from the wall's surface, i.e. the first and second adsorbate layer, is observed. For larger distances, the influence of the solid on the adsorbate layer is decreased to zero and the fluid-fluid interactions determine the upper levels of the adsorbate layer. Even though, a layering of the adsorbate layer is observed, its mole fraction for $y > 2.5\ \sigma$ is equal to the bulk liquid phase mole fraction calculated with the PeTS EOS. A rather similar behavior is observed for mixture III ($\xi_{\mathrm{AB}} = 1.25$, right bottom), where the fluid-fluid interactions determine the behavior of the upper levels of the adsorbate layer as well. However, for this mixture, the concentration changes from the bulk liquid phase mole fraction to the bulk vapor phase mole fraction from the 4th maximum of the density profiles to the bulk vapor phase. The same behavior can not be observed for mixture I, probably due to the smaller layer thickness and therefore, due to a superimposed influence of the solid on the adsorbate layer and the transition from the bulk liquid phase mole fraction to the bulk vapor phase mole fraction.

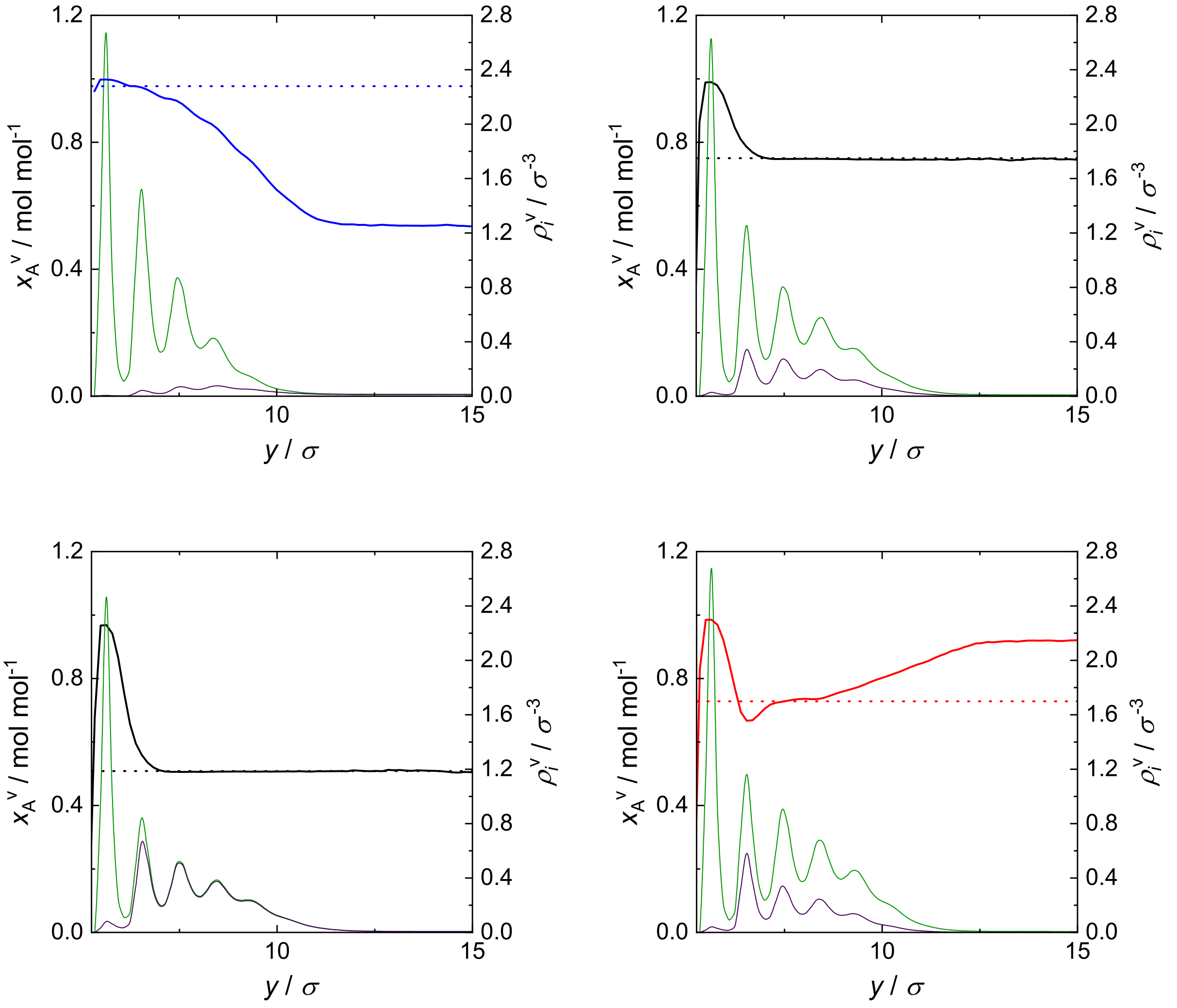

Figure S2: Concentration profiles $x_A^v(y)$ in the adsorbate layer for simulations with total wetting (thick solid lines) and corresponding liquid phase mole fraction of component A calculated with the PeTS EOS (thick dotted lines). The corresponding component density profiles of component A (green) and B (violet) are depicted as a guide. Left top: $\xi_{AB} = 0.7$ and $x''_A = 0.539$ mol mol^{-1} (blue); right top: $\xi_{AB} = 1.0$ and $x''_A = 0.508$ mol mol^{-1} (black); left bottom: $\xi_{AB} = 1.0$ and $x''_A = 0.750$ mol mol^{-1} (black); and right bottom: $\xi_{AB} = 1.25$ and $x''_A = 0.922$ mol mol^{-1} (red).
